# Differing pan-coronavirus antiviral potency of boceprevir and GC376 *in vitro* despite discordant molecular docking predictions

**DOI:** 10.1007/s00705-022-05369-y

**Published:** 2022-02-16

**Authors:** Yining Wang, Pengfei Li, Marla Lavrijsen, Yang Li, Zhongren Ma, Maikel P. Peppelenbosch, Mirza S. Baig, Qiuwei Pan

**Affiliations:** 1 Department of Gastroenterology and Hepatology, Erasmus MC-University Medical Center, room Na-1005, Wytemaweg 80, NL-3015, Rotterdam, CN The Netherlands; 2 Biomedical Research Center, Northwest Minzu University, Lanzhou, China; 3 Department of Biosciences and Biomedical Engineering (BSBE), Indian Institute of Technology Indore (IITI), Simrol, Indore, MP 453552 India

## Abstract

Given the structural similarities of the viral enzymes of different coronaviruses (CoVs), we investigated the potency of the anti-SARS-CoV-2 agents boceprevir and GC376 for counteracting seasonal coronavirus infections. In contrast to previous findings that both boceprevir and GC376 are potent inhibitors of the main protease (Mpro) of SARS-CoV-2, we found that GC376 is much more effective than boceprevir in inhibiting SARS-CoV-2 and three seasonal CoVs (NL63, 229E, and OC43) in cell culture models. However, these results are discordant with a molecular docking analysis that suggested comparable affinity of boceprevir and GC376 for the different Mpro enzymes of the four CoVs. Collectively, our results support future development of GC376 but not boceprevir (although it is an FDA-approved antiviral medication) as a pan-coronavirus antiviral agent. Furthermore, we caution against overinterpretation of *in silico* data when developing antiviral therapies.

**Supplementary Information:**

The online version contains supplementary material available at 10.1007/s00705-022-05369-y.

There are seven types of coronaviruses (CoVs) that are known to infect humans, including three highly pathogenic viruses – MERS-CoV, SARS-CoV-1, and SARS-CoV-2 – and four seasonal CoVs – NL63, 229E, OC43, and HKU1 – that usually, but not always, cause mild and self-limiting respiratory tract infections [1, 2]. Because of the ongoing COVID-19 pandemic, enormous efforts are being devoted to the quest for potent antiviral agents against SARS-CoV-2. One effective approach in this regard is targeting virus-encoded enzymes. The 5′-terminal region of human CoVs encodes the nonstructural proteins of these viruses, which include a 3-chymotrypsin-like protease (3CL or main protease), a papain-like protease (PLpro), a helicase, an RNA-dependent RNA polymerase (RdRp), an exoribonuclease and endoribonuclease, a methyl transferase, and several accessary proteins [3]. The main protease (Mpro) and PLpro are responsible for processing viral polypeptides, and Mpro has therefore become an appealing target for anti-SARS-CoV-2 drug development [4].

Through molecular docking and subsequent experimental validation, boceprevir and GC376 have recently been identified as potent inhibitors of SARS-CoV-2 Mpro [5, 6]. Boceprevir is a direct-acting antiviral medication that was approved by the FDA in 2011 for treatment hepatitis C virus (HCV) infection. It has been used extensively in the clinic and shows excellent anti-HCV efficacy with a favorable safety profile [5]. Thus, repurposing boceprevir for treating COVID-19 is relatively straightforward. In contrast, GC376 is a pre-clinical compound that has been shown to be effective against the coronavirus feline infectious peritonitis virus [7]. Thus, the development of GC376-based anti-SARS-CoV-2 therapy would require relatively extensive pre-clinical and clinical investigations, of its efficacy and safety.

Because of the structural similarities of the viral enzymes of different coronaviruses, we decided to explore the potential usefulness of established anti-SARS-CoV-2 agents for the treatment of seasonal coronavirus infections. Globally, seasonal coronaviruses cause approximately 5% of the several billion upper respiratory infections that occur each year and hence constitute an important health concern [2]. Furthermore, a subset of patients infected with a seasonal coronavirus can develop pneumonia [8], which can sometimes be fatal in vulnerable populations [9]. Among the four seasonal coronaviruses, NL63 is particularly interesting because it is the only seasonal coronavirus that, like SARS-CoV-1 and SARS-CoV-2, utilizes cellular angiotensin-converting enzyme 2 (ACE2) as its receptor for entry into its target cell [10]. NL63 was first isolated from a 7-month-old child suffering from bronchiolitis and conjunctivitis in the Netherlands [11]. We first evaluated the antiviral effect of a series of concentrations (0.1-300 μM) of boceprevir and GC376 in different cell models infected with NL63 (Fig. 1 and Supplementary Fig. S1). Unexpectedly, boceprevir and GC376 exhibited different levels of antiviral potency against NL63, although both inhibited viral replication in a dose-dependent manner in all of the cell models tested. The EC_50_ value of boceprevir was 36.31 μM, and that of GC376 was 0.7013 μM (Fig. 1A and B). When the potential therapeutic window for treating NL63 infection was calculated, we observed that the ratio of cytotoxic to antiviral activity was 17-fold higher for GC376 than for boceprevir (Fig. 1A and B; Supplementary Fig. S2A-F). Accordingly, the inhibitory effect of GC376 and boceprevir was significantly different for drug concentrations of 0.1, 1, 10, 30, and 100 μM (*P* < 0.01). For instance, treatment with 1 μM boceprevir had almost no antiviral effect in the Caco-2 cell model (a successful model system for studying drug effects on viral infection [12]), whereas treatment with 1 μM GC376 resulted in a 50% decrease in viral RNA levels (Fig. 1C). After 48-hours of drug treatment, supernatant samples from infected Caco-2 cells were collected, and the viral titer was determined using a TCID_50_ assay. We found that GC376, but not boceprevir, significantly reduces the viral titer of NL63 (Fig. 1D and E). This difference in antiviral potency was confirmed by quantifying secreted NL63 genomic RNA for 5 days from infected cells treated with 30 μM boceprevir or 1 μM GC376 (Fig. 1F and Supplementary Fig. S3).

**Fig. 1:**
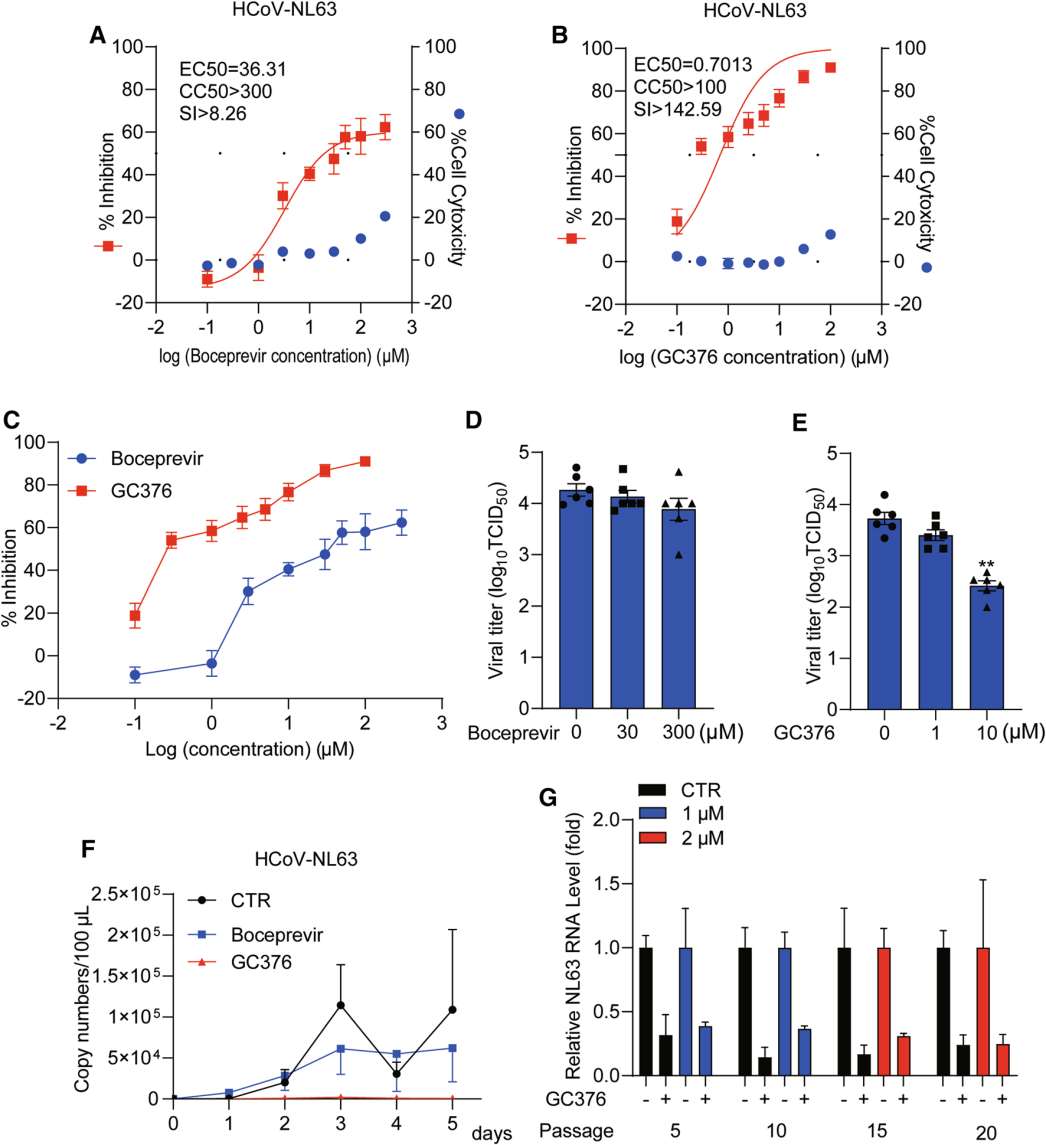
Antiviral effects of boceprevir and GC376 against the seasonal coronavirus NL63. (A and B) Caco-2 cells were infected with NL63 at an MOI of 0.1 and treated with different concentrations of boceprevir or GC376 for 48 hours. The viral yield in the cell supernatant was quantified by qRT-PCR. Cytotoxicity was determined by MTT assay. The left and right *y*-axes of the graphs represent mean % inhibition of virus yield and cytotoxicity of the drug, respectively (n = 6-16). (C) Comparison of viral RNA levels in the supernatant of infected Caco-2 cells treated with boceprevir or GC376. (D and E) Caco-2 cells were infected with NL63 at an MOI of 0.5 and treated with 30 or 300 μM boceprevir or 1 or 10 μM GC376 for 48 hours, and the virus titers were compared to those of untreated controls TCID_50_ assay (n = 6). (F) Caco-2 cells were infected with NL63 at an MOI of 0.5 and incubated for 5 days with 30 μM boceprevir or 1 μM GC376 or without an inhibitor. Supernatant was collected each day to quantify secreted viruses by qRT-PCR, calculated as the genomic copy number (n = 6). The standard curve for calculation of the genomic copy numbers is included in Supplementary Fig. S4. (F) NL63 was serially passaged in Caco-2 cells without GC376 (as a control) or with increasing concentrations of GC376 for 20 passages. 1 μM GC376 was used in passages 1-10, and the concentration was increased to 2 μM in the subsequent passages. The effect of GC376 (1 μM) on NL63 harvested at passage 5, 10, 15, and 20 was quantified using qRT-PCR. Data represent the mean ± SEM. *, *P* < 0.05; **, *P* < 0.01; ***, *P* < 0.001; HCoV, human coronavirus

Given the potent antiviral potency of GC376, we investigated the possible emergence of viral resistance following long-term treatment by serial passage of NL63 in the presence of increasing concentrations of GC376 and found that, after 20 passages, NL63 remained sensitive to GC376 treatment (Fig. 1G). These results are in accordance with previous findings that GC376 does not easily develop resistance and that the feline coronavirus 3CLpro has a high genetic barrier for selecting resistance against GC376 [13]. Of note, if Mpro inhibitors such as GC376 finally reach the clinic for treating coronavirus patients, it will be important to monitor the potential emergence of Mpro mutations, which may affect clinical response and treatment strategies.

Combination treatment is often used to enhance antiviral efficacy and to prevent the development of drug resistance in clinical applications. Remdesivir, the targeting viral RdRp, is the only FDA-approved antiviral drug for treating hospitalized COVID-19 patients [14]. Interferon alpha (IFN-α), a widely used antiviral drug, activates the host antiviral immune response [15] and has been clinically evaluated for treating COVID-19 patients [16]. We tested the combined effects of GC376 with remdesivir and GC376 with IFN-α by mathematical modeling using Synergy Finder [17]. Synergistic effects were observed with Loewe synergy scores of 17.509 ± 9.38 and 15.554 ± 11.84, respectively (Supplementary Fig. S4A-D). These antivirals were found to have little cytotoxicity in the host cells at the concentrations tested (Supplementary Fig. S4E).

Next, we investigated whether the difference in antiviral potency between boceprevir and GC376 would be observed with other coronaviruses. To this end, we used the human A549 lung cell line as an infection model for the seasonal coronaviruses 229E and OC43, and the Calu3 lung cell line was used for SARS-CoV-2 (Fig. 2 and Supplementary Fig. S5). Although dose-dependent inhibition of infection was observed with both agents, in accordance with the findings with NL63 (Fig. 1), GC376 was found to be much more potent against the other three human coronaviruses (Fig. 2 and Supplementary Fig. S5). The EC_50_ values for GC376 were below 3 μM for all three viruses, whereas for boceprevir they were all above 20 μM. Correspondingly, the selective indices for GC376 were much larger than those of boceprevir. For instance, in the SARS-CoV-2 model, the selective index of GC376 was over 150 but that of boceprevir was only about 10 (Fig. 2, Supplementary Fig. S2G-J, and Supplementary Fig. S5). Considering the global concerns about emerging SARS-CoV-2 variants, the delta variant in particular, we evaluated the antiviral effect of boceprevir and GC376 in Calu3 cells infected with the SARS-CoV-2 B.1.617.2 Delta variant. Consistent with the previous experiments, GC376 showed much more potent antiviral activity than boceprevir (Supplementary Fig. S6). Collectively, our results obtained using cell culture models demonstrate that GC376 is a much more potent inhibitor of different human coronaviruses than boceprevir.

**Fig. 2:**
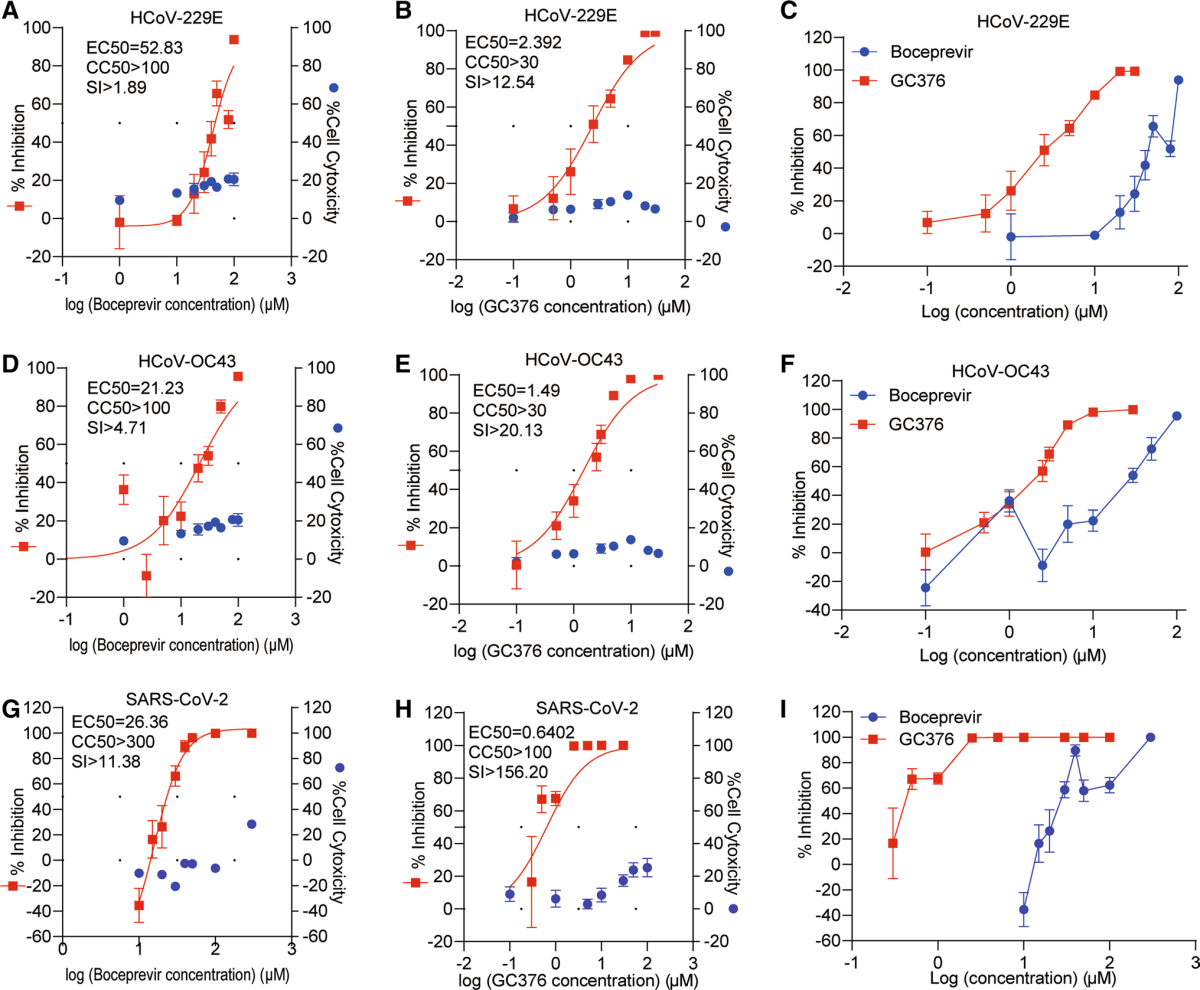
Antiviral effects of boceprevir or GC376 against 229E, OC43, and SARS-CoV-2 infection. (A, D, and G) A549 cells were infected with seasonal coronavirus 229E or OC43, and Calu-3 cells were infected with SARS-CoV-2 at an MOI of 0.1 and treated with different concentrations of boceprevir for 48 hours. The viral yield in the cell supernatant was then quantified by qRT-PCR. Cytotoxicity was determined by MTT assay. The left and right *y*-axes of the graphs represent the mean % inhibition of virus yield and cytotoxicity of the drug, respectively (n = 6-16). (B, E, and H) A549 cells were infected with 229E or OC43 and Calu-3 cells were infected with SARS-CoV-2 at an MOI of 0.1 and treated with different concentrations of GC376 for 48 hours. The viral yield in the cell supernatant was then quantified by qRT-PCR. Cytotoxicity was determined by MTT assay. The left and right *y*-axes of the graphs represent mean % inhibition of virus yield and cytotoxicity of the drugs, respectively (n = 6-16). (C, F, and I) Comparison of the inhibition of 229E, OC43, and SARS-CoV-2 replication by boceprevir and GC376 treatment. Data represent the mean ± SEM. *, *P* < 0.05; **, *P* < 0.01; ***, *P* < 0.001; HCoV, human coronavirus

Previously, boceprevir and GC376 were both predicted to be potent inhibitors of the SARS-CoV-2 Mpro by *in silico* docking analysis [5, 6]. We therefore performed a molecular docking simulation for these two agents using molecular models of the Mpro proteins of the coronaviruses NL63, 229E, OC43, and SARS-CoV-2. We used existing crystal structures of Mpro to model the ones for which experimentally determined structures are not yet available (see detailed methods in the supplementary file) (Fig. 3 and Supplementary Fig. S7). Based on their docking scores, boceprevir and GC376 were predicted to have similar binding affinity for each of the four coronavirus Mpro (Fig. 3). We suggest two possible explanations for the disparity observed between molecular docking and cell culture experiments. First, although the binding energy (Fig. 3) is comparable, the binding modes of boceprevir and GC376 might be very different in relation to antiviral activity. As shown in Fig. 3, there are clearly different patterns for the two drugs with respect to the drug-interacting regions, the numbers and positions of hydrogen bonds predicted to form, and the van der Waals interactions expected. However, it is not known how these different interaction patterns might result in the functional differences in antiviral activity between boceprevir and GC376 observed in our *in vitro* experimentation. Second, both agents could have similar ability to inhibit Mpro, but GC376 might have additional antiviral properties that are yet unknown.

**Fig. 3:**
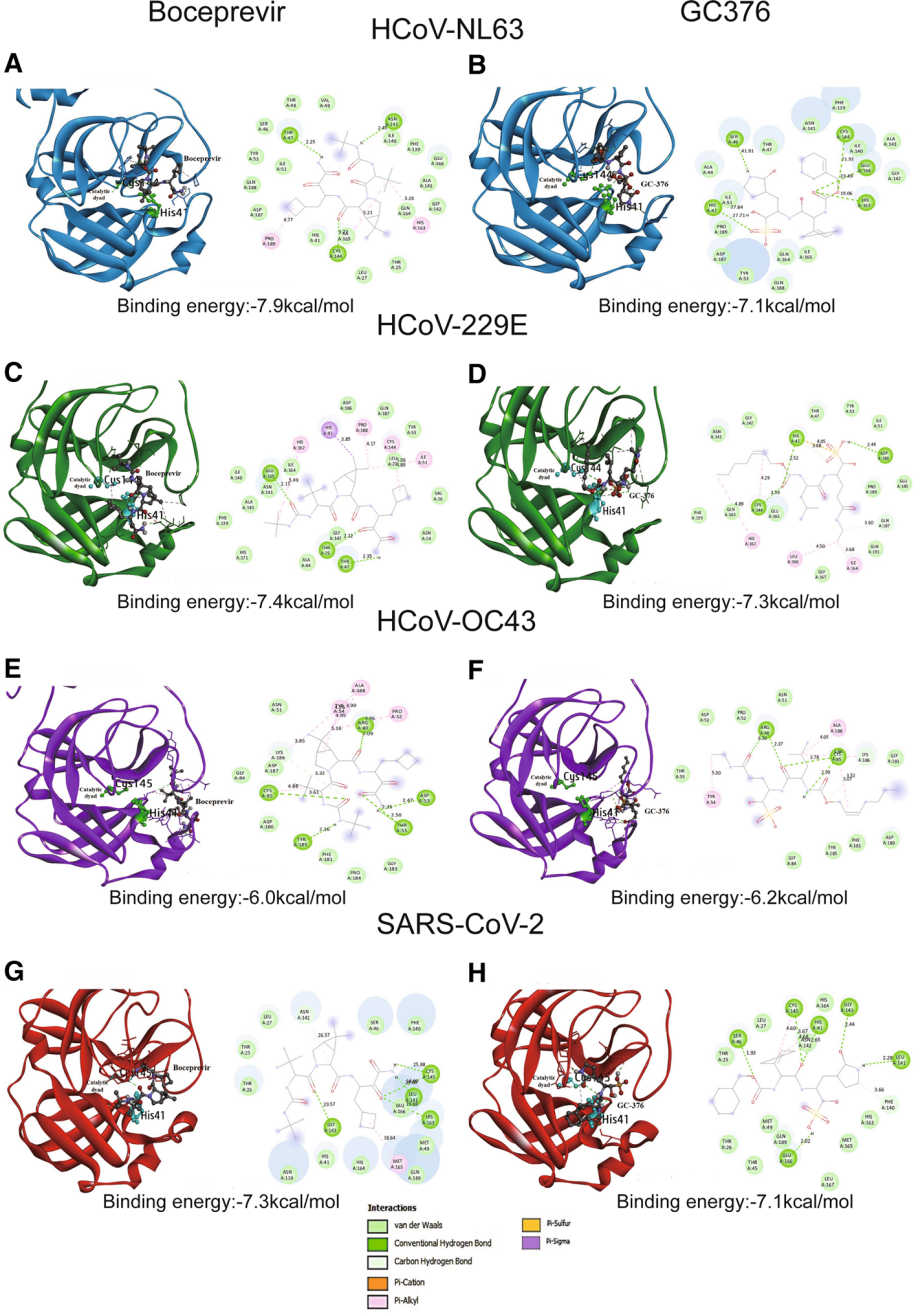
Site-specific binding mode of boceprevir and GC376 to coronavirus Mpro. Boceprevir, binding to the Mpro (ribbons colored according to atom type) of NL63 (A), 229E (C), OC43 (E), and SARS-CoV-2 (G), is depicted as a surface representation. GC376, binding to the Mpro (ribbons colored according to atom type) of NL63 (B), 229E (D), OC43 (F), and SARS-CoV-2 (H), is depicted as a surface representation. Binding energy is indicated in kcal/mol. H-bond donor (purple) and acceptor (green) interactions are shown. HCoV, human coronavirus

In summary, although molecular docking predicted similar affinity of boceprevir and GC376 towards the Mpro enzymes of different coronaviruses, their actual antiviral activity in cell culture models differed. Our results, which partially contradict previous findings, support future development of GC376 but not boceprevir as a pan-coronavirus antiviral agent. We are currently unable to explain the mechanism underlying the disparities between the molecular docking and cell culture results, but we urge caution in the interpretation of *in silico* data when developing antiviral therapies.

## Supplementary Information

Below is the link to the electronic supplementary material.

Supplementary file1 (PDF 1253 kb)
