# Supplementary material for: Differing pan-coronavirus antiviral potency of boceprevir and GC376 in vitro despite discordant molecular docking predictions
Source: Arch Virol. 2022 Feb 16;167(4):1125–30. doi: 10.1007/s00705-022-05369-y (PMC8853085; doi:10.1007/s00705-022-05369-y)
Supplement: Supplementary file 1 — Supplementary file1 (PDF 1253 kb) [file 705_2022_5369_MOESM1_ESM.pdf]

Supplementary information to

Distinct pan-coronavirus antiviral potency of boceprevir and GC376 in cell culture models in discordance with molecular docking

Yining Wang[a], Pengfei Li[a], Marla Lavrijsen[a], Yang Li[a], Zhongren Ma[c], Maikel P Peppelenbosch[a], Mirza S. Baig[b]*, Qiuwei Pan[a]*

a. Department of Gastroenterology and Hepatology, Erasmus MC-University Medical Center, Rotterdam, The Netherlands

b. Department of Biosciences and Biomedical Engineering (BSBE), Indian Institute of Technology Indore (IITI), Simrol, Indore, India.

c Biomedical Research Center, Northwest Minzu University, Lanzhou, China

Supplementary Materials and methods

Reagents and antibodies

Boceprevir (Sanbio, The Netherlands) and GC376 (Sigma-Aldrich, The Netherlands) were dissolved in dimethyl sulfoxide (DMSO, Sigma, Zwijndrecht, The Netherlands). Remdesivir (MedChem Express, USA) and human IFN-α (Sigma-Aldrich, The Netherlands) were dissolved in phosphate-buffered saline (PBS).

Viruses and cell lines

Monkey LLCMK-2 cells were cultured in minimal essential medium with Earle's

salt (MEM; Gibco, Grand Island, USA) containing 8% (vol/vol) heat-inactivated fetal calf serum (FCS, Sigma–Aldrich, St. Louis USA), 1% (vol/vol) nonessential amino acid (Sciencell, San Diego, California, USA), 0.1% (vol/vol) L-Glutamine (Lonza, Verviers, Belgium), 100 IU/mL Penicillin and 100 mg/mL Streptomycin (Gibco, Grand Island, USA). Multiple cell lines including human colon cancer cell line Caco-2, human hepatoma cell line Huh7, monkey kidney cell line Vero-E6 and adenocarcinomic human alveolar basal epithelial cell line A549 were cultured with Dulbecco's modifified Eagle medium (DMEM) (Lonza Biowhittaker, Verviers, Belgium) supplemented with 10% (vol/vol) heat-inactivated fetal calf serum (FCS, Sigma–Aldrich, St. Louis USA), 100 IU/mL Penicillin and 100 mg/mL Streptomycin (Gibco, Grand Island, USA). Human lung cancer cell line Calu-3 were cultured in advanced DMEM/F12 supplemented with 1% (vol/vol) GlutaMAX^{TM} Supplement (Gibco, Grand island, USA), 10 mM HEPES. Human seasonal coronavirus NL63 stock was produced by consecutively inoculating the virus onto LLCMK-2 cells. Seasonal coronavirus OC43 and 229E were bought from ATCC (USA) and amplified in Huh7 cells. SARS-CoV-2 (isolate BetaCoV/Munich/BavPat1/2020; European Virus Archive Global #026V-03883, GenBank: MT270101) and SARS-CoV-2 B.1.617.2 Delta variant (isolated from a patient) were kindly provided by Dr. Bart Haagmans (Department of Viroscience, Erasmus MC). Cell lines were analyzed by genotyping and confirmed to be mycoplasma negative.

Virus production and inoculation re-infection assay

LLCMK-2 cells harboring the infectious NL63 were seeded into multi-well plates, culturing at 33 °C, with 5% CO_2 for 5-7 days, over 50% of cells have cytopathic effect (CPE), then harvested NL63 particles by repeated freezing and thawing three times, filtered with 0.45 μm filters. Huh7 cells harboring the infectious OC43 or 229E were seeded into multi-well plates, culturing at 33 °C, with 5% CO_2 for 4-6 days. When over 50% of cells have cytopathic effect (CPE), OC43 or 229E particles were harvested by repeated freezing and

thawing three times, then filtered with 0.45 μm filters. SARS-CoV-2 stocks were produced as previously described [1]. In short, Vero-E6 cells harboring the infectious SARS-CoV-2 were seeded into multi-well plates and incubating the cells at 37 °C, with 5% CO_2 for 72 hours. The culture supernatant was cleared by centrifugation and stored in aliquots at −80°C. Cells were seeded into multi-well plates and culture medium was discarded when cell confluence was approximately 80%, followed by twice washing with 1×PBS. Harvested NL63 viruses were added, and incubated overnight. Harvested OC43 or 229E viruses were added, incubated for 2 h at 33 °C. Harvested SARS-CoV-2 viruses were added, incubated for 1 h at 37 °C, with 5% CO_2, followed by three times washing with 1×PBS to remove unattached viruses. Then cells were incubated with culture medium for another 48 hours. The virus titers were analyzed by TCID50 assay.

Antiviral drug treatment

LLCMK-2, Huh7 and Caco-2 cells were first inoculated with NL63 at a multiplicity of infection (MOI) of 0.1, and incubated at 33 °C overnight, A549 cells were first inoculated with 229E or OC43 at a MOI of 0.1, and incubated at 33 °C 2 h, and Calu-3 cells were first inoculated with SARS-CoV-2 or SARS-CoV-2 B.1.617.2 Delta variant at a MOI of 0.1 or 0.01, and incubated at 37 °C for 1 hour. The cells were then washed twice with PBS to remove free virus particles and treated with boceprevir or GC376 for the indicated time period. Cells, total RNA or supernatant were collected for further analysis.

RNA isolation, cDNA synthesis and qRT-PCR

Total RNA was isolated using Macherey-Nagel NucleoSpin® RNA II kit (Bioke, Leiden, The Netherlands) and quantified using a Nanodrop ND-1000 (Wilmington, DE, USA). cDNA was synthesized by using a cDNA synthesis kit (TaKaRa Bio, Inc., Shiga, Japan). Real-time PCR reactions were performed with SYBR-Green-based real-time PCR (Applied Biosystems®, Austin, USA)

on a StepOnePlusTM System (Thermo Fisher Scientific LifeSciences). Glyceraldehyde 3-phosphate dehydrogenase (GAPDH) gene was used as housekeeping gene. Relative gene expression of target gene was normalized to GAPDH using the formula $2^{-\Delta\Delta CT}$, $\Delta\Delta CT = \Delta CT sample - \Delta CT control$ (ΔCT = CT [target gene] - CT[GAPDH]). Template control and reverse transcriptase control were included in all qRT-PCR experiments, and all primers are listed in Supplementary table 1.

MTT assay

LLCMK-2, Huh7, Caco-2, A549, Calu-3 cells were seeded into 96-well tissue culture plates (1×10^4cells/well), and then treated with the indicated compounds for 48 hours. Cells were incubated with 10 µL 5 mg/mL 3-(4,5-dimethyl-2-thiazolyl) -2,5-diphenyl-2H-tetrazolium bromide (MTT) for 3 hours, then replaced with 100 µL DMSO medium and incubated at 37°C for 30 minutes. The absorbance at 490 nm was recorded using a microplate absorbance reader (Bio-Rad, CA, USA).

TCID50 assay

Viruses in the cultured cells the supernatant were harvested through repeated freezing and thawing for three times. NL63 titer was quantified by using a 50% tissue culture infectious dose (TCID50) assay. Briefly, ten-fold dilutions of NL63 were inoculated onto LLCMK-2 cells, grown in a 96-well tissue culture plate at 2,000 cells/well. The plate was incubated at 33 °C for 7 days, and each well was examined under a light microscope for cytopathic effect (CPE). The TCID50 value was calculated by using the Reed-Muench method.

Quantification of NL63 genome copy numbers

An amplicon of NL63 (a fragment of N protein) were cloned into the pCR2.1-TOPO vector (Invitrogen, San Diego, CA) to generate a template for quantifying NL63 genome copy numbers. The plasmid was extracted by Quick

Plasmid Miniprep Kit (Invitrogen, Lohne, Germany). A series of dilutions (from 10^{-1} to 10^{-8}) were prepared and then were amplified and quantified by qRT-PCR to generate a standard curve. This standard curve was generated by plotting the log copy number versus the cycle threshold (CT) value (Fig. S3). Copy numbers were calculated by using the following equation: Copy number (molecules/μl) = [concentration (ng/μL) × 6.022× 10^{23} (molecules/mol)]/ [length of amplicon× 640 (g=/mol) × 10^{9} (ng/g)].

Serial passaging of NL63 with GC376 treatment

NL63 was passaged in Caco-2 cells in the absence of drug (vehicle control) or in the presence of gradually increasing concentrations of the drug (1 μM of GC376 for passage 1–10 and 2 μM of GC376 for passage 11–20). In brief, Caco2 cells in 6-well plate were inoculated with virus (MOI = 0.5) at 33 °C for overnight, followed by adding GC376 or without drug (as control). After 48 hours, both cells and supernatant were harvested, subsequently frozen, thawed once, and centrifuged. The supernatant containing passaged viruses was stored at −80 °C until used for the next passage. Viruses were serially passaged by using 1 aliquot of viral stock from the preceding passage to infect fresh Caco2 cells. The effect of each passage of virus (same titer) was quantified by qRT-PCR.

3D protein structure modeling of coronavirus Mpro

The experimentally solved crystal structure of SARS-CoV-2 Mpro (PDB Id 6M2Q), NL63 Mpro (PDB Id 5GWY), and 229E Mpro (PDB Id 2ZU2) were retrieved from RCSB PDB database (https://www.rcsb.org). The retrieved structures were prepared for docking in Discovery Studio Visualizer. The hetero atoms, water molecules and any co-crystalized ligand groups were removed followed by addition of polar hydrogen atoms. The crystal structure for OC43 Mpro was not available at PDB database, therefore, we perform the homology-based 3D structure modelling in the Modeller 10.1 tool [2]. Briefly,

the protein sequence of OC43 Mpro was submitted for structure-based similarity search in NCBI-BLAST using RCSB PDB as the search database to obtain best template for modelling and HKU1 Mpro (PDB ID: 3D23) showed a percent identity of 82% with the sequence of OC43 Mpro as well as 99% query cover for structure modelling. A total of 50 models were prepared in Modeller 10.1 and were ranked based on their DOPE (Discrete Optimized Protein Energy) score. After loop refinement of the top model, the stereochemical properties of final structure was analyzed in SAVES v6.0 (https://saves.mbi.ucla.edu) which showed the Ramachandran value of 95.5% amino acids in the most favored region while no residues in disallowed region and an ERRAT (quality factor) score of 97%, demonstrating a good model structure for further study. Lastly, the root mean square deviation was also computed between the template (HKU1 Mpro) and the OC43 modelled structure by superimposing in Chimera tool and we obtained a RMSD value of 0.398Å [3] (Fig. S7).

Protein-ligand docking

Boceprevir (CID 10324367) and GC-376 (CID 71481120) retrieved from PubChem database were prepared for molecular docking with Mpro structures of four coronaviruses. The protein-ligand docking was performed in AutoDock Vina and top most pose (1st docking pose) with highest binding energy (in kcal/mol) was opted for further analysis [4]. In addition to binding energy, it was very important to analyze the interaction of both drugs with residues of Mpro, mainly their non-covalent intermolecular interaction in the catalytic target site. The interaction analysis between drugs and protein was performed in Discovery studio visualizer.

Statistics analysis

All numerical results were reported as Mean ± SEM. The statistical significance of differences between means was assessed with the

Mann-Whitney test (GraphPad Prism 5; GraphPad Software Inc., La Jolla, CA). The threshold for statistical significance was defined as $P \leq 0.05$.

Supplementary Table 1. Primers used in the study

Gene name	F-sequence (5' to 3')	R-sequence (5' to 3')
HCoV-NL63 plasmid	CTTCTGGTGACGCTAGTACAGCTTAT	AGACGTCGTTGTAGATCCCTAACAT
Human GAPDH	GTCTCCTCTGACTTCAACAGCG	ACCACCCTGTTGCTGTAGTAGCCA A
NL63	ACGCAATGCCACTGTTGTTA	GACAACACCGTCATCAGAGA
229E	GTCGTCAGGGTAGAATACCTTA	CCCGTTTGCGCTTTCTAGT
OC43	AGCAACCAGGCTGATGTCAATACC	AGCAGACCTTCCTGAGCCTTCAAT
SARS-CoV-2	CAATGGTTTAACAGGCACAGG	CTCAAGTGTCTGTGGATCACG

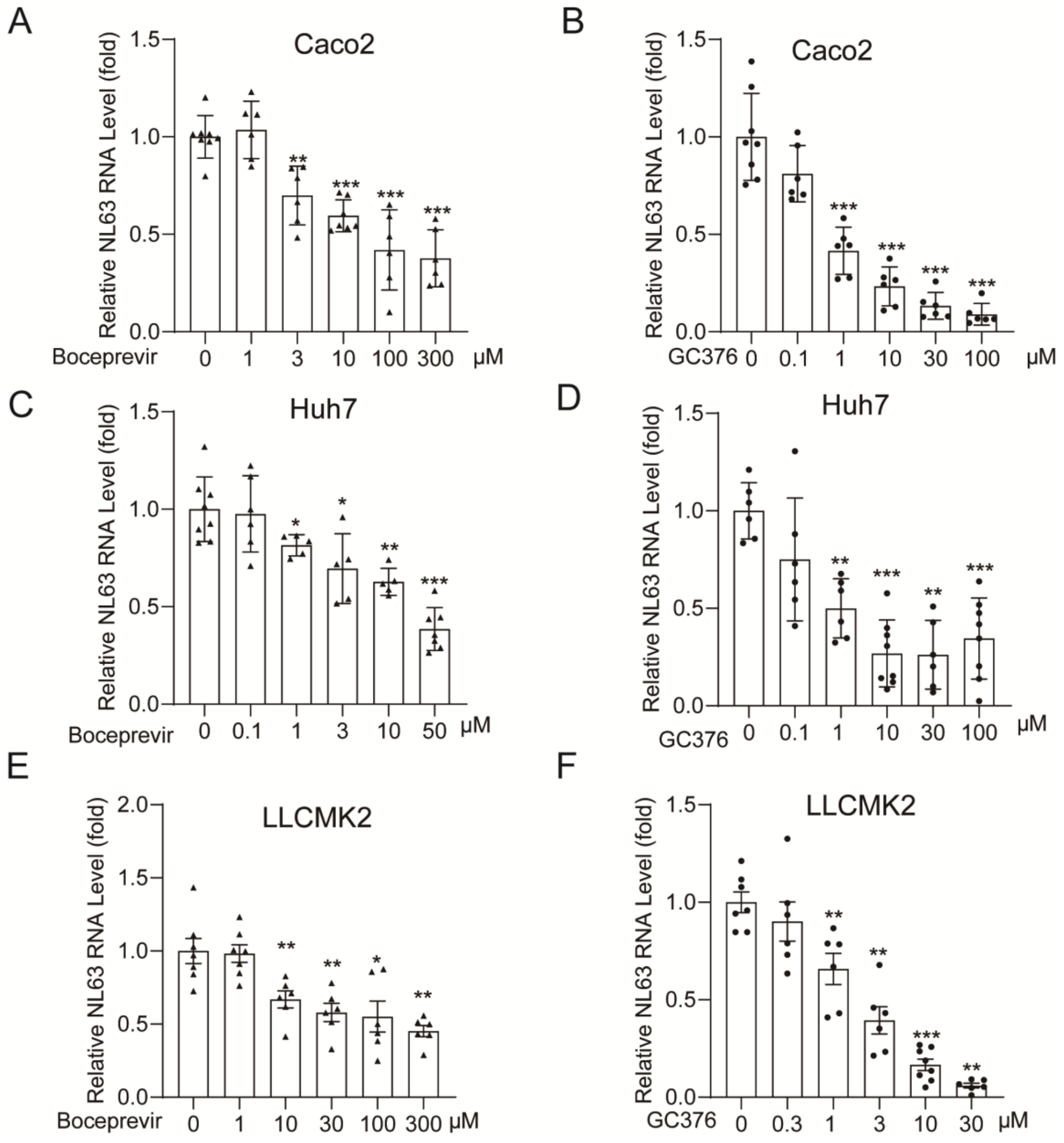

Supplementary Fig. S1. **Antiviral effects of boceprevir and GC376 against NL63 in different cell culture models.** (A), (C) and (E) Dose-dependent inhibition of HCoV-NL63 replication in Caco-2, Huh7 and LLCMK-2 cell lines by boceprevir treatment. Intracellular viral RNA quantified by qRT-PCR was normalized to housekeeping gene GAPDH and presented relative to the control (CTR) (set as 1) (n = 5-8). (B), (D) and (F) Dose-dependent inhibition of NL63 replication in Caco-2, Huh7 and LLCMK-2 cell lines by GC376 treatment.

Intracellular viral RNA quantified by qRT-PCR was normalized to housekeeping gene GAPDH and presented relative to the control (CTR) (set as 1) ($n = 6$-8). Data represent as mean ± SEM. *$P < 0.05$; **$P < 0.01$; ***$P < 0.001$.

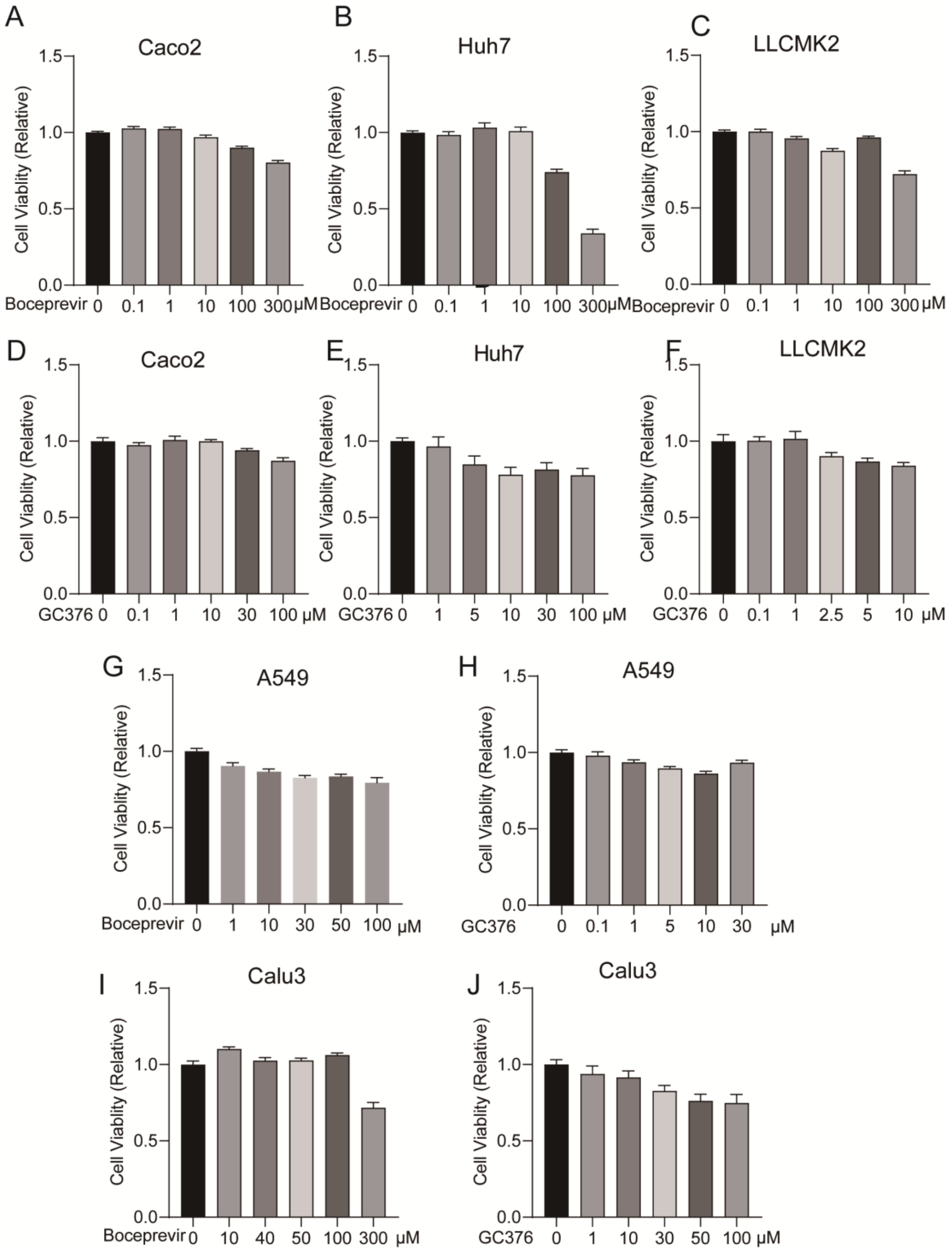

Supplementary Fig. S2. The cytotoxicity of boceprevir and GC376 on different cell lines. (A), (B), (C), (G) and (I) Caco-2, Huh7, LLCMK-2, A549 and Calu-3 cells treated with different concentrations of boceprevir for 48 hours. Cytotoxicity was determined by MTT assay (n = 10-16). (D), (E), (F), (H) and (J)

Caco-2, Huh7, LLCMK-2, A549 and Calu-3 cells treated with different concentrations of GC376 for 48 hours. Cytotoxicity was determined by MTT assay (n = 10-16).

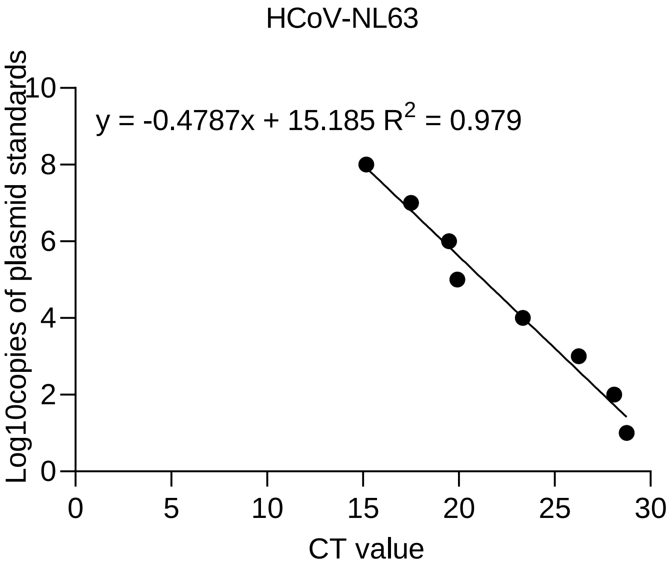

Supplementary Fig. S3. Standard curve for quantifying NL63 genome copy numbers. Amplicon of the N protein of NL63 was cloned into the pCR2.1-TOPO vector. The plasmid was extracted, followed by a series of dilutions from 10^{-1} to 10^{-8} and then were amplified and quantified by qRT-PCR. Standard curve was generated by plotting the cycle threshold (CT) value regarding the log copy numbers.

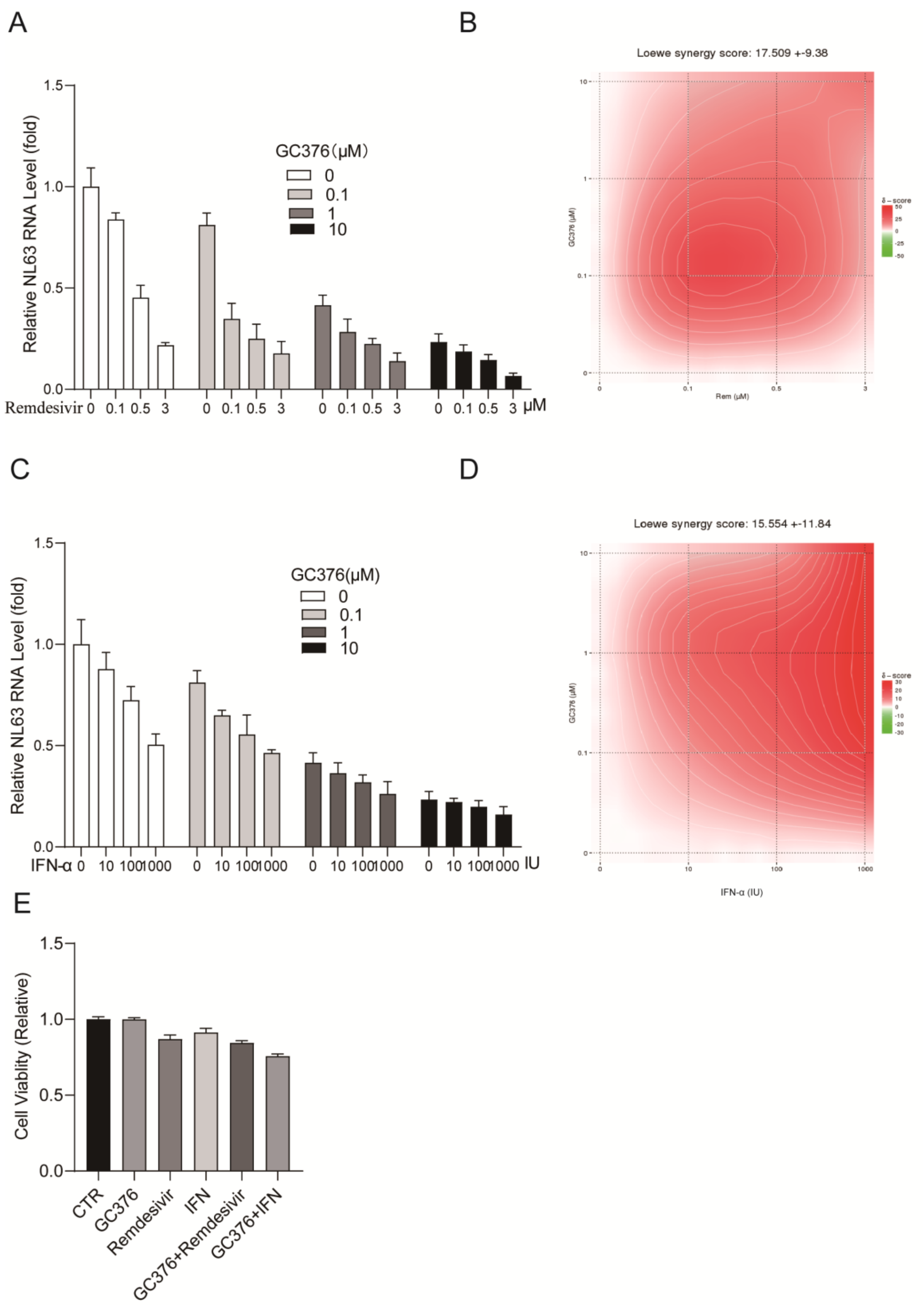

Supplementary Fig. S4. The effects of combining GC376 with remdesivir or IFN-α on NL63 infection. (A) and (C) The antiviral effects of combining various

concentrations of GC376 with remdesivir or IFN-α. Data represent as mean ± SEM. *P < 0.05; **P < 0.01; ***P < 0.001. (B) and (D) Synergy plot representing the score of antiviral activity for the GC376-remdesivir or GC376-IFN-α combination based on the data shown in A and C (n = 4-6). (E) Cytotoxicity of GC376, remdesivir or IFN-α and their combinations. Caco-2 cells were treated with different concentrations of GC376 (10 µM), remdesivir (3 µM), IFN-α (1000IU), or their combinations respectively for 48 h. Cytotoxicity was determined by MTT assay (n = 8-16). HCoV: human coronavirus

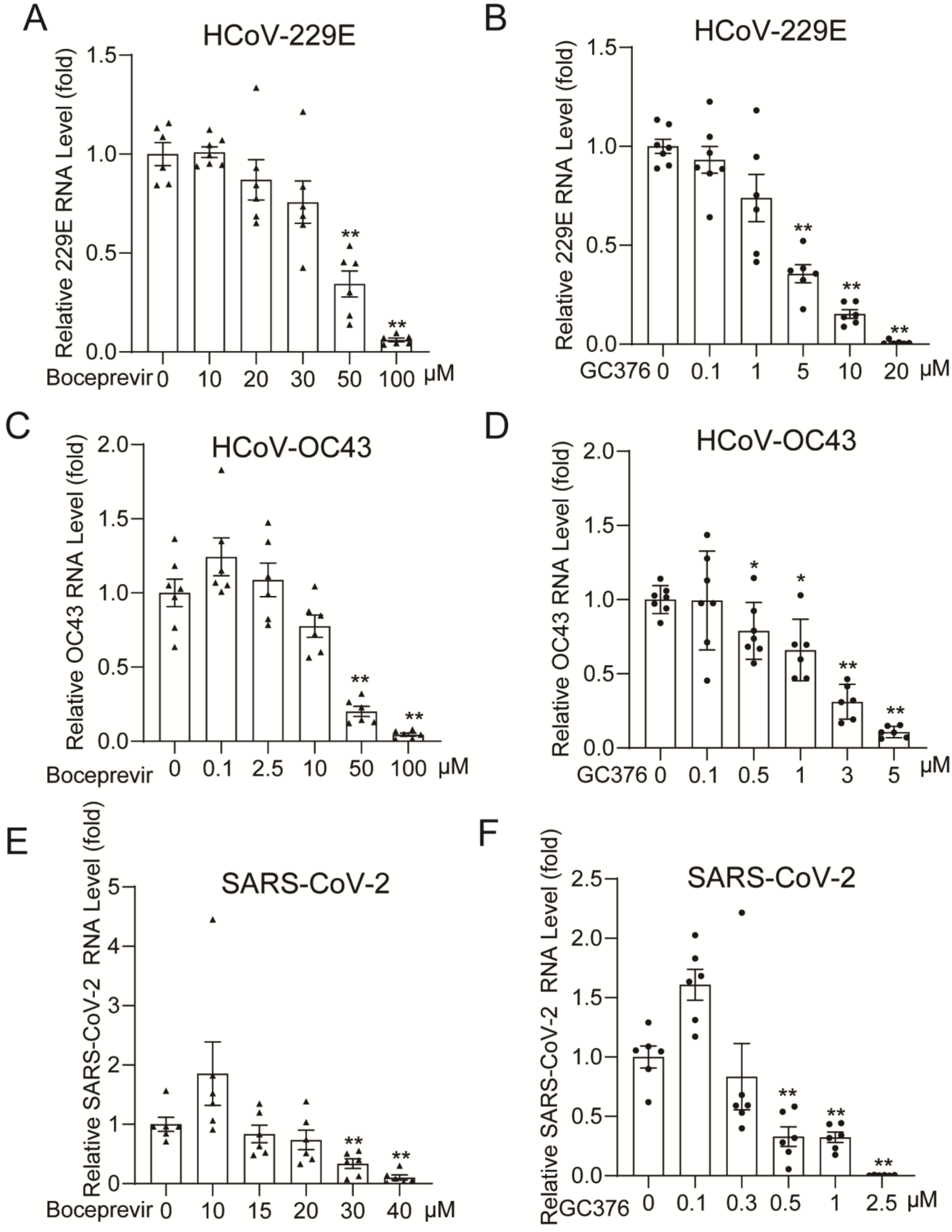

Supplementary Fig. S5. Antiviral effects of boceprevir and GC376 against

229E, OC43 and SARS-CoV-2. (A), (C) and (E) Dose-dependent inhibition of 229E, OC43 and SARS-CoV-2 replication by boceprevir treatment. Intracellular viral RNA quantified by qRT-PCR was normalized to housekeeping gene GAPDH and presented relative to the control (CTR) (set as 1) (n = 6-7). (B), (D) and (F) Dose-dependent inhibition of 229E, OC43 and SARS-CoV-2 replication by GC376 treatment. Intracellular viral RNA quantified by qRT-PCR was normalized to housekeeping gene GAPDH and presented relative to the control (CTR) (set as 1) (n = 6-7). Data represent as mean ± SEM. *P < 0.05; **P < 0.01; ***P < 0.001.

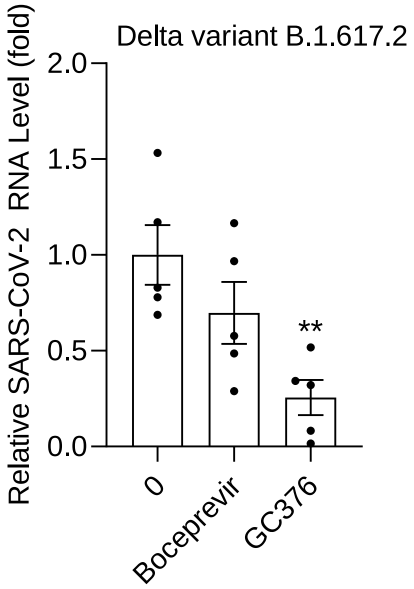

Supplementary Fig. S6. Antiviral effects of boceprevir and GC376 against SARS-CoV-2 B.1.617.2 Delta variant. Inhibition of SARS-CoV-2 B.1.617.2 Delta variant replication by 3 μM boceprevir or GC376 treatment. Intracellular viral RNA quantified by qRT-PCR was normalized to housekeeping gene GAPDH and presented relative to the control (CTR) (set as 1) (n = 5). Data represent as mean ± SEM. *P < 0.05; **P < 0.01; ***P < 0.001.

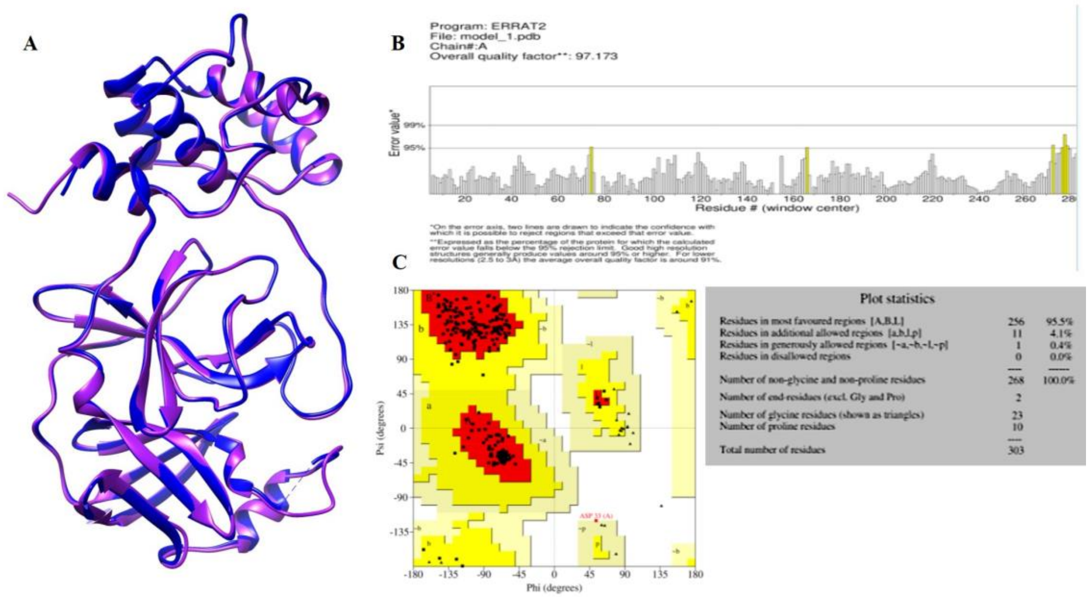

Supplementary Fig. S7. Homology-based 3D structure modelling of OC43 Mpro in Modeller 10.1 tool. The OC43 Mpro was modelled from HKU1 (PDB Id 3D23) structure as template. (A) The root mean square deviation (RMSD) between template (blue) and model structure (purple) was computed in chimera tool as 0.398Å only, (B) An ERRAT score of 97% provides an overall quality factor, and (C) The Ramachadaran plot shows 95.5% amino acid in most favored region while no residues in disallowed region stereo chemically signifies the quality of OC43 Mpro modelled structure.

References

1. Lamers MM, Beumer J, van der Vaart J, Knoops K, Puschhof J, Breugem TI, Ravelli RBG, Paul van Schayck J, Mykytyn AZ, Duimel HQ, van Donselaar E, Riesebosch S, Kuijpers HJH, Schipper D, van de Wetering WJ, de Graaf M, Koopmans M, Cuppen E, Peters PJ, Haagmans BL, Clevers H (2020) SARS-CoV-2 productively infects human gut enterocytes. Science 369:50-54

2. Sali A, Blundell TL (1993) Comparative protein modelling by satisfaction of spatial restraints. J Mol Biol 234:779-815

3. Pettersen EF, Goddard TD, Huang CC, Couch GS, Greenblatt DM, Meng EC, Ferrin TE (2004) UCSF Chimera--a visualization system for exploratory research and analysis. J Comput Chem 25:1605-1612

4. Trott O, Olson AJ (2010) AutoDock Vina: improving the speed and accuracy of docking with a new scoring function, efficient optimization, and multithreading. J Comput Chem 31:455-461
